# Germline *EMSY* sequence alterations in hereditary breast cancer and ovarian cancer families

**DOI:** 10.1186/s12885-017-3488-x

**Published:** 2017-07-24

**Authors:** Kirsi M. Määttä, Riikka Nurminen, Minna Kankuri-Tammilehto, Anne Kallioniemi, Satu-Leena Laasanen, Johanna Schleutker

**Affiliations:** 1 Institute of Biosciences and Medical Technology - BioMediTech, University of Tampere, Lääkärinkatu 1, FI-33520 Tampere, Finland; 2 Fimlab Laboratories, Tampere University Hospital, Biokatu 4, FI-33520 Tampere, Finland; 3 Department of Clinical Genetics, Turku University Hospital, Kiinamyllynkatu 4-8, FI-20521 Turku, Finland; 4 Department of Pediatrics, Genetics Outpatient Clinic, and Department of Dermatology, Tampere UniversityHospital, PO BOX 2000, FI-33521 Tampere, Finland; 5 Department of Dermatology, Tampere University Hospital, PO BOX 2000, FI-33521 Tampere, Finland; 6 Institute of Biomedicine, University of Turku, Kiinamyllynkatu 10, FI-20014 Turku, Finland; 7 Department of Medical Genetics, Turku University Hospital, Kiinamyllynkatu 10, FI-20521 Turku, Finland

**Keywords:** Breast cancer, Ovarian cancer, Germline, *EMSY*

## Abstract

**Background:**

*BRCA1* and *BRCA2* mutations explain approximately one-fifth of the inherited susceptibility in high-risk Finnish hereditary breast and ovarian cancer (HBOC) families. *EMSY* is located in the breast cancer-associated chromosomal region 11q13. The *EMSY* gene encodes a BRCA2-interacting protein that has been implicated in DNA damage repair and genomic instability. We analysed the role of germline *EMSY* variation in breast/ovarian cancer predisposition. The present study describes the first *EMSY* screening in patients with high familial risk for this disease.

**Methods:**

Index individuals from 71 high-risk, *BRCA1/2*-negative HBOC families were screened for germline *EMSY* sequence alterations in protein coding regions and exon-intron boundaries using Sanger sequencing and TaqMan assays. The identified variants were further screened in 36 Finnish HBOC patients and 904 controls. Moreover, one novel intronic deletion was screened in a cohort of 404 breast cancer patients unselected for family history. Haplotype block structure and the association of haplotypes with breast/ovarian cancer were analysed using Haploview. The functionality of the identified variants was predicted using Haploreg, RegulomeDB, Human Splicing Finder, and Pathogenic-or-Not-Pipeline 2.

**Results:**

Altogether, 12 germline *EMSY* variants were observed. Two alterations were located in the coding region, five alterations were intronic, and five alterations were located in the 3'untranslated region (UTR). Variant frequencies did not significantly differ between cases and controls. The novel variant, c.2709 + 122delT, was detected in 1 out of 107 (0.9%) breast cancer patients, and the carrier showed a bilateral form of the disease. The deletion was absent in 897 controls (OR = 25.28; *P* = 0.1) and in 404 breast cancer patients unselected for family history. No haplotype was identified to increase the risk of breast/ovarian cancer. Functional analyses suggested that variants, particularly in the 3'UTR, were located within regulatory elements. The novel deletion was predicted to affect splicing regulatory elements.

**Conclusions:**

These results suggest that the identified *EMSY* variants are likely neutral at the population level. However, these variants may contribute to breast/ovarian cancer risk in single families. Additional analyses are warranted for rare novel intronic deletions and the 3'UTR variants predicted to have functional roles.

**Electronic supplementary material:**

The online version of this article (doi:10.1186/s12885-017-3488-x) contains supplementary material, which is available to authorized users.

## Background

Breast cancer (BC) is the most common cancer among women in Western countries. In Finland, 4694 new BC cases were diagnosed in 2012 (Finnish Cancer Registry). Ovarian cancer (OC) is the most lethal gynaecologic malignancy in developed countries [1]. In 2012, 466 new OC cases were diagnosed, making OC the tenth most common cancer among Finnish women (Finnish Cancer Registry). The genetic predisposition to both of these diseases has been well recognized. Approximately 5–10% of all of breast and ovarian cancer cases reflect inherited genetic defects primarily in two well-known high-penetrance breast and ovarian cancer genes, *BRCA1 (breast cancer 1, early onset)* and *BRCA2 (breast cancer 2, early onset)* [2–4]. Both of these genes encode large proteins that play critical roles in the DNA repair pathway (reviewed in [5]). Mutations in *BRCA1* and *BRCA2* predispose to hereditary breast and ovarian cancer (HBOC) syndrome characterized by multiple family members affected with breast or ovarian cancer or both, early onset of BC, bilateral form of cancer, and the appearance of other cancers in family members, including prostate, pancreatic and male BC [6]. Among Finnish HBOC families, *BRCA1/2* mutations are explain approximately 20% [7, 8]. Additionally, a low proportion of additional HBOC predisposition in the Finnish families reflects defects in other DNA repair pathway genes, including *CHEK2 (Checkpoint kinase 2), PALB2 (Partner and localizer of BRCA2), RAD51C (RAD51 (S. cerevisiae) Homologue C)*, and *Abraxas* [9–12]. Nevertheless, additional HBOC predisposing genetic factors remains unknown, and one potential approach is to screen the candidate genes of proteins that interact with either *BRCA1* or *BRCA2* in the DNA repair pathway.

One of the interacting proteins of BRCA2 is EMSY (C11orf30) [13]. *EMSY* is located at chromosomal region 11q13 (11q13.5), which is associated with BC, particularly the hormone receptor-positive form of the disease [14]. *EMSY* encodes a protein with an evolutionary conserved EMSY N-terminal domain that is unique in the human genome [13]. The EMSY N-terminal domain has a structure similar to the DNA-binding motif found in transcription factors [15].

EMSY has been implicated in DNA repair and transcriptional regulation. The interaction with BRCA2 correlates EMSY to DNA repair with the observation that EMSY localizes at DNA damage sites [13]. In addition, the over-expression of a truncated form of EMSY results in a chromosome instability phenotype in human mammary epithelial cells, similar to that of cells showing a loss of BRCA2 function [16]. The binding of EMSY to the BRCA2 exon 3-encoding transcriptional activation domain represses the function of this domain [13]. In addition, EMSY interacts with the chromatin remodelling proteins Heterochromatin protein 1 β (HP1 β) and BS69 [13]. EMSY has been implicated in the regulation of nuclear receptor-mediated transcription [17], the transcription of interferon-stimulated genes with BRCA2 [18], and the transcription of antimetastatic microRNA miR-31 [19].

*EMSY* is amplified in breast tumours [13, 20–23], and this amplification is associated with the poor outcome of BC [13, 20–22]. *EMSY* copy number changes have also been observed in male breast tumours, but the copy number gains are comparatively more frequent in female breast tumours [24]. In addition to breast cancer, EMSY is overexpressed in high-grade ovarian cancer [13, 25, 26] and pancreatic cancer [27]. Previously, we examined the association of *EMSY* single-nucleotide polymorphisms (SNPs) in relation to prostate cancer predisposition and identified a rare intronic SNP that increases the risk of aggressive prostate cancer [28].

In the present study, we identified germline sequence alterations in the *EMSY* gene, encoding a BRCA2-interacting protein partner, which could contribute to HBOC susceptibility by disrupting critical functions in the DNA repair pathway. Thus, we sequenced the *EMSY* coding region and exon-intron boundaries in a cohort of 71 Finnish *BRCA1/2*-negative HBOC patients pre-screened for mutations in seven known breast cancer genes and copy-number alterations at the genome-wide scale [29, 30] and further analysed the identified variants in additional HBOC patients and healthy controls. To our knowledge, the present study is the first to analyse germline *EMSY* alterations in susceptibility to HBOC in high-risk families.

## Methods

### Patients and controls

Germline *EMSY* sequence alterations were screened in index patients from 71 high-risk Finnish HBOC families. Study material was collected from the Tampere University Hospital Genetics Outpatient Clinic between January 1997 and May 2008. Patients belonging to a cohort of 82 high-risk HBOC individuals previously well characterized and screened for germline alterations in *BRCA1, BRCA2, CHEK2, PALB2, BRIP1, RAD50,* and *CDH1* genes and germline copy number alterations at the genome-wide scale [29, 30]. From the previously described cohort, we included only the 71 affected index individuals in the present study, including 57 females with BC, 8 females with bilateral BC, 1 female with OC, and 5 females with both BC and OC. Originally, all patients were determined as negative for 28 Finnish *BRCA1/2* founder-mutations based on minisequencing and protein truncation tests (PTTs) for *BRCA1* exon 11 and *BRCA2* exons 10 and 11. Additionally, the exons and exon-intron boundaries of *BRCA1* and *BRCA2* have previously been analysed using Sanger sequencing and Multiplex Ligation-dependent Probe amplification (MLPA) to exclude other mutations [29]. All of the identified *EMSY* sequence alterations were screened from the DNA samples of 36 additional index patients from HBOC families collected from the Turku region and from anonymous healthy female blood donors (*n* = 380–904) (referred as controls) obtained from the Finnish Red Cross. Additionally, a rare novel c.2709 + 122delT variant was further screened in a cohort of 404 BC cases unselected for family history from the Tampere region [31]. All patients were informed of the analyses and provided written consent to the use of existing DNA samples in the present study. The Ethical Committees of Tampere and Turku University Hospitals and the National Authority for Medicolegal Affairs approved this research project.

### Sample preparation and mutation screening

Genomic DNA from control samples was extracted from peripheral leukocytes using the Puregene Kit according to the manufacturer’s instructions (Gentra Systems, Inc., Minneapolis, MN, USA). Mutation screening was achieved through Sanger sequencing. Reference sequence NM_020193.3 was obtained from the USCS genome browser [32, 33]. The genome build GRCh37 (hg19) was used. Whole coding regions and exon-intron boundaries were analysed. *EMSY* primer sequences and PCR conditions are available upon request. Sequencing was performed using the Big Dye Terminator v.3.1 Cycle Sequencing Kit and the ABIPRISM 3130xl Genetic Analyser (Applied Biosystems, Foster City, CA, USA). The sequences were analysed using Sequencher v.5.1 software (Gene Codes Corporation, Ann Arbor, MI, USA). The RefSNP number for the identified variants was obtained from the NCBI Single Nucleotide Polymorphism database (dbSNP) [34]. Two variants, rs1044265 and rs2513513, were analysed from controls using TaqMan SNP Genotyping Assays according to the manufacturer’s instructions with the ABI Prism® 7900HT instrument and SDS2.2.2 software (Applied Biosystems). The genotyping call rates of the SNPs were ≥0.98.

### Statistical and bioinformatics analyses

Observed variants were tested for Hardy-Weinberg equilibrium in the controls. The association of variants with breast/ovarian cancer was examined using Fisher’s exact test. *P* values were two-sided, and *P* < 0.05 was considered statistically significant. The association of the minor allele was examined. Odds ratios (OR) and confidence intervals (CI) were calculated using PLINK v1.07 [35]. If the variant was not observed in cases or controls, then the OR was calculated using GraphPad Prism version 5.02 for Windows (GraphPad Software, San Diego, CA, USA) by adding 0.5 to each value to obtain numeric values. Linkage disequilibria (LD) between variants, haplotype blocks and the association of haplotypes with breast/ovarian cancer were analysed using Haploview v4.2 [36]. Haplotype blocks were defined using 107 cases and 380 controls via Gabriel’s method, which includes by default SNPs with minor allele frequencies (MAFs) > 0.05 [37].

The effect of the amino-acid changing variant, c.2861 T > G (Leu954Arg), was predicted using the Pathogenic-or-Not-Pipeline (PON-P2) programme [38]. The functionality of the observed SNPs with rs IDs was analysed using HaploReg v2 [39], including conserved region, open chromatin, regulatory chromatin state from the Encyclopaedia of DNA Elements (ENCODE) [40], protein binding, and altered motifs. The four alternative positions of the deletion c.2709 + 122delT (chr11 76,253,530–76,253,533) were analysed for functional elements using RegulomeDB [41], which includes data for predicted and known regulatory elements, such as regions of DNA hypersensitivity, binding sites of transcription factors, and regions with enhancer activity.

The effect of the deletion c.2709 + 122delT on splicing was analysed using Human Splicing Finder (HSF) v2.4.1 [42]. The alternative deletion locations were analysed using HSF to detect potential splice sites (HSF matrices), potential branch points, enhancer motifs (ESE Finder) and hnRNP motifs (experimental) of SR proteins.

## Results

In the present study, we identified 12 different germline *EMSY* sequence alterations in index patients from 71 HBOC families, and the alterations were further screened in a cohort of 36 HBOC patients and healthy controls. Variant frequencies are presented in Table 1. Two of the identified alterations were located in the protein-coding region, five alterations were intronic, and five variants were located in the 3′UTR. With the exception of one alteration, c.2709 + 122delT, all of the identified variants are reported in the NCBI Single Nucleotide Polymorphism (SNP) database with rs-numbers (Table 1).

**Table 1:** Identified *EMSY* sequence alterations

Nucleotide change	Amino acid change	Position	rs number	Genotype distribution *n* (%)^a^		*P*-value	OR; 95% CI
				Cases	Controls		
c.1108 + 40A > G	−	76,183,924	rs4245443	16/49/42 (15.0/45.8/39.3)	54/182/137 (14.5/48.8/36.7)	0.8	0.96; 0.70–1.31
c.1514-4G > A	−	76,227,182	rs2508740	15/50/42 (14.0/46.7/39.3)	44/178/149 (11.9/48.0/40.1)	0.7	1.07; 0.78–1.46
c.1685-14C > T	−	76,234,185	rs11600501	103/4/0 (96.3/3.7/0)	366/11/0 (97.1/2.9/0)	0.8	1.29; 0.41–4.08
c.1995 + 47delA	−	76,237,726	rs11363199	49/44/14 (45.8/41.1/13.1)	168/168/41 (44.6/44.6/10.8)	0.9	1.02; 0.74–1.41
c.2709 + 122delT	−	76,253,530–76,253,533	−	106/1/0 (99.1/0.9/0)	897/0/0 (100/0/0)	0.1	25.28; 1.02–625.0
c.2861 T > G	p.Leu954Arg	76,255,454	rs184345272	104/2/0 (98.1/1.9/0)	376/3/0 (99.2/0.8/0)	0.3	2.40; 0.40–14.44
c.3648 T > C	p.Thr1216Thr	76,257,215	rs3753051	49/44/14 (45.8/41.1/13.1)	166/165/41/ (44.6/44.4/11.0)	0.9	1.02; 0.74–1.41
c.*343A > G	−	76,261,533	rs2513513	16/45/46 (15.0/42.1/43.0)	55/186/139 (14.5/48.9/36.6)	0.5	0.88; 0.64–1.21
c.*631C > G	−	76,261,821	rs148932730	105/2/0 (98.1/1.9/0)	370/5/0 (98.7/1.3/0)	0.7	1.41; 0.27–7.30
c.*744A > C	−	76,261,934	rs187735484	98/9/0 (91.6/8.4/0)	331/45/0 (88.0/12.0/0)	0.4	0.69; 0.33–1.44
c.*753G > C	−	76,261,943	rs72932407	99/8/0 (92.5/7.5/0)	351/27/0 (92.9/7.1/0)	0.8	1.05; 0.47–2.34
c.*938A > G^b^	−	76,262,128	rs1044265	38/51/18 (35.5/47.7/16.8)	351/417/128 (39.2/46.5/14.3)	0.4	1.14; 0.85–1.52

*CI* confidence interval, *OR* odds ratio

^a^Genotype distribution denotes homozygotes/heterozygotes/homozygotes in the order indicated in the nucleotide change column. In the case of deletion, genotype distribution denotes no deletion/heterozygous deletion/homozygous deletion

^b^Due to the Hardy-Weinberg disequilibrium in 380 controls, the variant was genotyped in a larger number of controls

*3'UTR variant, i.e. the variant is downstream (3') of the translation termination site (http://varnomen.hgvs.org/bg-material/numbering/)

All SNPs were in Hardy Weinberg equilibrium in controls. The allele frequencies did not significantly differ between cases and controls (Table 1). One of the SNPs, rs2508740, was triallelic. Interestingly, the T allele, observed in one control sample, has not previously been reported in the SNP database. However, this control sample was not included in the association testing of the SNP. One of the two variants observed in the protein-coding region rs184345272 resulted in an amino acid substitution of a hydrophobic leucine with a positively charged arginine at position 954. The heterozygous rs184345272 variant was identified in two patients (2 of 106, 1.9%) and three controls (3 of 376, 0.8%) (OR = 2.40; *P* = 0.3). PON-P2 predicts the unknown effects of substitutions.

**Fig. 1:**
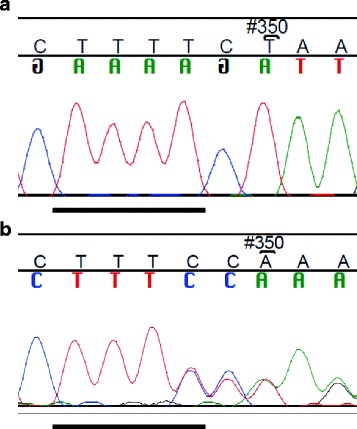
The novel germline sequence alteration c.2709 + 122delT in *EMSY*. **a** Control sample sequence without the deletion. **b** Heterozygous deletion in a BC patient sample. The precise position of the deleted T is uncertain (the region where the T has been deleted is *underlined in black* in both **a** and **b**)

The most interesting finding was the rare novel sequence alteration, c.2709 + 122delT (Fig. 1). The precise location of the deleted T could not be determined based on the sequence because of a stretch of four T bases in the reference sequence (chr11 76,253,530–76,253,533). This deletion has been named according to the Human Genome Variation Society (HGVS) nomenclature (http://varnomen.hgvs.org, v.15.11). The heterozygous deletion was identified in one patient (1 of 107, 0.9%) with bilateral BC diagnosed at 39 and 42 years of age. However, none of the controls (0 of 897, 0%) had this deletion (OR = 25.28; *P* = 0.1) (Table 1), and it was not observed in a cohort of 404 BC cases unselected for family history (data not shown). The deletion carrier patient died of BC at age 52. The clinical features of the patient included invasive ductal type grade 1 tumour in the left breast and invasive ductal type grade 2 tumour in the right breast. Both the tumours had oestrogen and progesterone receptor positive and human epidermal growth factor receptor 2 negative statuses. The patient’s mother had BC diagnosed at age 51, and the father had pancreatic cancer diagnosed at age 64. The patient had one healthy brother. Additionally, the deletion carrier patient had four other heterozygous *EMSY* variants, rs42445443, rs2508740, rs2513513, and rs1044265.

**Fig. 2:**
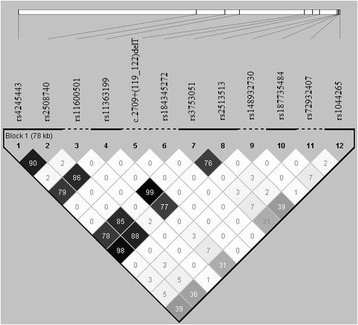
The haplotype block structure. Haplotype analysis included the genotypes of the observed variants from 107 breast and/or ovarian cancer cases and 380 controls. The first alternative marker position was used for c.2079 + 122delT. Linkage disequilibrium (LD) values (r^2^ × 100) are represented in the chart in *shades of black*. The figure was obtained using Haploview [36]

Based on haplotype block analysis, seven observed variants (rs4245443, rs2508740, rs11363199, rs3753051, rs2513513, rs187735484 and rs1044265) formed a haplotype block (Fig. 2). A total of six haplotypes were observed. The haplotype AAATAAA was more common in controls (2.6%) than cases (0.5%) with a borderline significance difference (*P* = 0.05). The SNP rs2508740 was in high LD (r^2^ ≥ 0.8) with rs3753051, rs11363199, rs2513513, and rs4245443 (Fig. 2). In addition, high LD was observed for SNPs rs4245443 and rs2513513 and SNPs rs11363199 and rs3753051 (Fig. 2).

The functionality of the identified SNPs with rs IDs (all but c.2709 + 122delT) was analysed in silico using HaploReg v.2 [39] (Table 2). Some differences were observed in conserved regions between GERP and SiPhy approaches, but the conserved regions primarily overlapped with the SNPs located in exonic and 3′UTR regions. The 3′UTR SNPs were located within open chromatin in several cell lines and coincided with the chromatin states of strong enhancers. In addition, two intronic variants (rs11600501 and rs11363199) were located within chromatin states of weak enhancers. Nine of the 11 analysed variants were predicted to affect regulatory motifs. None of the variants were located within protein binding sites.

**Table 2:** Functional annotation of the identified *EMSY* SNPs with rs IDs

SNP	Location	Conserved region		Open chromatin^a^;	Regulatory chromatin state^a^;	Protein binding	Motifs changed
		GERP	SiPhy	Cell ID^b^	(Cell ID^b^)		
rs4245443	intron	No	No	–	–	–	Pou1f1_2, YY1_known6
rs2508740	intron	No	Yes	–	–	–	–
rs11600501	intron	Yes	Yes	–	7_Weak_Enhancer (HepG2)	–	EWSR1-FLI1, GATA_known8,
							TATA_disc7
rs11363199	intron	No	No	–	7_Weak_Enhancer (HepG2)	–	Foxa_known4, Pou1f1_2,
							Pou2f2_known2, Pou3f2_2, Sox_5
rs184345272	exon	Yes	Yes	–	–	–	NRSF_known3
rs3753051	exon	Yes	Yes	HeLa-S3	–	–	CEBPB_disc2, Foxa_known2
rs2513513	3′UTR	Yes	Yes	HA-sp., HCPEpiC,	5_Strong_Enhancer (Huvec)	–	–
				HMVEC-dBl-Neo			
rs148932730	3′UTR	Yes	Yes	–	4_Strong_Enhancer (Huvec)	–	DMRT2, Nanog_disc2, Sox_13,
							Sox_14, Sox_16, Sox_18, Sox_19,
							Sox_2, Sox_4, Sox_9
rs187735484	3′UTR	Yes	No	HMVEC-LBl,	4_Strong_Enhancer (Huvec)	–	TATA_disc7
				HMVEC-dLy-Neo			
rs72932407	3′UTR	Yes	No	HBMEC, HMVEC-LBl,	4_Strong_Enhancer (Huvec)	–	MAZ, SREBP_known3
				HMVEC-dLy-Neo,			
				HPAEC, HRGEC			
rs1044265	3′UTR	Yes	Yes	HUVEC	5_Strong_Enhancer (Huvec)	–	Foxo_3, Mef2_known1, Mef2_known6,
							Pou2f2_known2, TCF4_known3

^a^The ENCODE data [40]

^b^ *HeLa-S3* cervical carcinoma, *HA-sp.* astrocytes spinal cord, *HCPEpiC* choroid plexus epithelial cells, *HMVEC-dBl-Neo* neonatal blood microvascular endothelial cells, dermal-derived, *HMVEC-LBl* blood microvascular endothelial cells, lung-derived, *HMVEC-dLy-Neo* neonatal lymphatic microvascular endothelial cells, dermal-derived, *HBMEC* brain microvascular endothelial cells, *HPAEC* pulmonary artery endothelial cells, *HRGEC* renal glomerular endothelial cells, *HUVEC* umbilical vein endothelial cells, *HepG2* hepatocellular carcinoma

The functionality of the c.2709 + 122delT variant was examined using the Regulome DB [41], and the four alternative locations of the deletion coincided with the same functional elements, including the open chromatin and transcription factor binding site of GATA6 (Additional file 1: Table S1). In addition, the four-base location coincides with three regulation motif positions.

The effect of c.2709 + 122delT on splicing regulatory elements was analysed using Human Splicing Finder [42] (Additional file 1: Table S2), which indicates both the introduction and deletion of an acceptor splice site as a result of the deletion of either one of the alternative nucleotides. The deletion of the third or fourth nucleotide introduced and abolished a potential branch point. No enhancer motifs of SR proteins or silencer motifs of hnRNP were affected by this deletion.

## Discussion

Genetic factors predisposing to breast and ovarian cancer primarily remain unknown in HBOC families negative for *BRCA1* and *BRCA2* mutations. Here, we screened the *EMSY* gene, encoding the BRCA2-interacting protein, for germline sequence alterations and analysed the association of the observed variants with breast/ovarian cancer risk. We utilized a cohort of index individuals from 71 high-risk *BRCA1/2*-negative HBOC families previously screened for germline alterations in seven known BC genes and copy number alterations at the genome-wide scale [29, 30]. According to previous analyses, no known predisposing variants have been identified in a majority (87%) of the screened high-risk families [29, 30], indicating the existence of yet unknown gene variants contributing to breast/ovarian cancer susceptibility. To our knowledge, this study is the first to screen the *EMSY* gene for germline variations in relation to breast/ovarian cancer in high-risk families.

We identified 12 different variants in the coding regions and exon-intron boundaries, but none of these variants showed a statistically significant association with breast/ovarian cancer risk at the population level, which may reflect the limited sample size in the present study. Moreover, haplotype analysis identified one haplotype as more common in controls compared to cases but with borderline significance difference. However, the identified variants, particularly the rare novel deletion, c.2709 + 122delT, could be important predisposing factors in individual patients and in a few cancer families. Interestingly, the deletion carrier patient was affected with bilateral BC, which may indicate that this deletion contributes to a poor clinical outcome of the disease. The deletion carrier patient did not have deleterious mutations or copy number changes in the previously screened BC genes, *BRCA1*, *BRCA2*, *CHEK2*, *PALB2, BRIP1*, *RAD50*, and *CDH1* [29, 30]. Unfortunately, we did not obtain blood samples from the relatives of the deletion carrier patient to examine the segregation of the deletion with the disease. Although this deletion is located in an intronic region, it may represent a functional variant, according to the results of the in silico functional analyses. Thus, further studies of this deletion are warranted. Of note, during the review process of this manuscript, the deletion was published in the dbSNP and received reference SNP id number 983125332.

Only one missense variant (rs184345272) was identified in the protein-coding region of *EMSY*, suggesting that mutations are either rare or not tolerated. The rs184345272 variant, predicted to have unknown effect on protein function, was 2.4 times more common in cases compared to controls, but obviously a larger number of samples should be screened to determine whether this variant could be a low-risk allele. Interestingly, five of the 12 (41.6%) alterations occurred in the 3′UTR and were predicted to play functional roles. Further screening of these variants would be interesting, particularly for the rs148932730 variant, which was detected as 1.4 times more common in cases vs. controls.

No truncating mutations, such as frameshift or nonsense mutations, were detected. Because *EMSY* is amplified or over-expressed in breast and ovarian cancer tumours [13, 20–23, 25, 26], predisposing germline alterations are expected to result in the gain-of-function rather than the loss-of-function of *EMSY*. Based on the in silico functional annotation, either of the alternatives can be ruled out, since, for example, the potential effect of the c.2709 + 122delT deletion on splicing might affect the function of *EMSY*. Unfortunately, tumour DNA was not available from the deletion patient for additional analysis, for example, loss of heterozygosity (LOH). Therefore, further functional studies are needed to clarify these findings and characterize the effects of the variants on the functions of other genes through gene regulation.

Common *EMSY* variations associated with breast and ovarian cancer risks have previously been examined in British population-based studies [43]. Three out of the six SNPs observed in the British study [43] (rs4245443, rs2508740, and rs11600501) were also identified in the cohort in the present study. We did not observe an association of these SNPs with breast/ovarian cancer risk, consistent with previous results [43].

We previously examined the association of *EMSY* SNPs with prostate cancer predisposition and identified a rare intronic SNP that increases the risk of aggressive prostate cancer (referred to as aggressive SNP) [28]. Interestingly, the aggressive SNP was segregated with breast cancer in a prostate cancer family [28]. However, in the present study, we did not detect the aggressive SNP. Since we only screened one individual per breast/ovarian cancer family, we cannot rule out that the other affected family members in the examined cohort could be carriers of the previously detected aggressive SNP.

## Conclusions

In conclusion, this study is the first to analyse the role of germline *EMSY* sequence alterations in HBOC predisposition in Finnish families. None of the observed *EMSY* variants showed statistically significant associations with breast/ovarian cancer risk at the population level. Based on the results, we cannot exclude the likelihood variants contributing to cancer risk in certain high-risk families, perhaps as private mutations. The variants could be used to determine cancer risk with other predisposing mutations in these families. Further analyses are warranted, particularly for the rare novel intronic deletion and 3′UTR variants predicted to have functional roles.

## Additional file

Additional file 1: Table S1. Functional annotation of the location of the c.2709 + 122delT deletion. **Table S2**. The effect of the c.2709 + 122delT variant on splicing regulatory elements. (DOCX 16 kb)

## Abbreviations

BC: Breast cancer
*BRCA1*: *Breast cancer 1, early onset*
*BRCA2*: *Breast cancer 2, early onset*
*CHEK2*: *Checkpoint kinase*
CI: Confidence interval
HBOC: Hereditary breast and ovarian cancer
HGVS: Human Genome Variation Society
HP1 β: Heterochromatin protein 1 β
LD: Linkage disequilibria
LOH: Loss of heterozygosity
MAF: Minor allele frequency
MLPA: Multiplex Ligation-dependent Probe amplification
OC: Ovarian cancer
OR: Odds ratio
*PALB2*: *Partner and localizer of BRCA2*
PTT: Protein truncation test
*RAD51C*: *RAD51 (S. cerevisiae) Homologue C*
SNP: Single-nucleotide polymorphism
UTR: Untranslated region
