# Supplementary material for: Germline EMSY sequence alterations in hereditary breast cancer and ovarian cancer families
Source: BMC Cancer. 2017 Jul 24;17:496. doi: 10.1186/s12885-017-3488-x (PMC5525221; doi:10.1186/s12885-017-3488-x)
Supplement: Additional file 1: Table S1. — Functional annotation of the location of the c.2709 + 122delT deletion. Table S2. The effect of the c.2709 + 122delT variant on splicing regulatory elements. (DOCX 16 kb) [file 12885_2017_3488_MOESM1_ESM.docx]

**Additional file 1.**

**Table S1. Functional annotation of the location of the deletion c.2709+122delT.**

|  |  |  |
| --- | --- | --- |
| **Functional element^a^** | **Cell ID^b^** | **Protein** |
| Open chromatin |  |  |
| DNase-seq | Panc1 | - |
| FAIRE | K562 | - |
| Protein binding | Caco2 | GATA6 |
| Motifs changed | - | STAT1, STAT5B (homodimer), STAT1 |
| ^a^ The four alternative positions (chr11 76253530-76253533) of the deletion gave similar results. | | |
| ^b^ Panc1 = pancreatic carcinoma; K562 = leukemia; Caco2 = colorectal adenocarcinoma | | |

**Table S2. The effect of the c.2709+122delT variant on splicing regulatory elements.**

|  |  |
| --- | --- |
| **Effect on regulatory element^a^** | **Alternative location** |
| A less strong acceptor splice site introduced (56.4 → 78.9) | 1, 2, 3, 4 |
| A strong acceptor splice site broken (82.8 → 26.5) | 1, 2, 3, 4 |
| A potential branch point introduced (43.8 → 77.3) | 3, 4 |
| A potential branch point broken (80.6 → 0^b^, 12.1^c^) | 3, 4 |
| ^a^ The consensus values (CV) of motifs are reported in brackets.  ^b^ The value of the third alternative location. ^c^ The value of the fourth alternative location | |
